# Amyotrophic Lateral Sclerosis (ALS) Genetics and Microbiota: A Comprehensive Review

**DOI:** 10.3390/ijms27041978

**Published:** 2026-02-19

**Authors:** Mostafa Ahmed Kurdi, Hidayah Alotaibi, Asayel Tawfiq Alkhuraymi, Layyan Nassar Aldahery, Ali Fouad Alhawaj, Hamzah Jehad Aldali

**Affiliations:** 1 Biology Department, College of Science, Taibah University, Medina 42353, Saudi Arabia; 2 Department of Biology, College of Science, Princess Nourah bint Abdulrahman University, Riyadh 11671, Saudi Arabia; 3 Biology Department, Faculty of Science, King Abdul Aziz University, Jeddah 21589, Saudi Arabia; 4 Department of Physiology, College of Medicine, Imam Abdulrahman Bin Faisal University, Dammam 34212, Saudi Arabia; 5 Cellular and Molecular Medicine, College of Biomedical Science, University of Bristol, Bristol BS8 1DT, UK

**Keywords:** amyotrophic lateral sclerosis, genetics, microbiota

## Abstract

Amyotrophic Lateral Sclerosis (ALS) is a severe, progressive neurodegenerative disorder characterized by the loss of upper and lower motor neurons, affecting 0.5 to 2.6 per 100,000 people, with a median survival of 2 to 5 years. It is increasingly seen as a multisystem disorder, sharing essential clinicopathological features with Frontotemporal Dementia (FTD). This convergence arises from overlapping molecular processes, including severe oxidative stress, glutamate-mediated excitotoxicity, mitochondrial dysfunction, and widespread aggregated TDP-43 proteinopathy in both sporadic and familial cases. Several key genetic factors have been identified, particularly mutations in *C9orf72*, *SOD1*, *TARDBP*, and *FUS*, which serve as important targets for novel treatments, such as Tofersen, a recently approved SOD1-specific antisense oligonucleotide (ASO) gene therapy. Additionally, there is increasing evidence of the gut–brain connection. Dysbiosis, involving species such as *Akkermansia muciniphila,* and lower levels of neuroprotective metabolites, such as nicotinamide, may affect the course of the disease. As a result, treatment strategies are shifting toward a personalized approach. This includes using gene therapy, ranging from ASOs and RNA interference (RNAi) to new CRISPR-based genome editing. It also involves exploring microbiome-modulating treatments, such as specific probiotics and Fecal Microbiota Transplantation (FMT). While microbiome and gene therapies remain largely experimental, their potential is promising, as highlighted by the recent approval of Tofersen. These novel approaches could be further enhanced and guided by more robust diagnostic criteria and by investigating early multimodal treatment strategies to slow the progression of this complex disease.

## 1. Introduction to ALS 

Amyotrophic lateral sclerosis (ALS), commonly referred to as Lou Gehrig’s disease, is a progressive and ultimately fatal neurodegenerative disorder characterized by the selective degeneration of both upper motor neurons (UMN) and lower motor neurons (LMN) in the brainstem and spinal cord [1,2]. This dual involvement results in a combination of spasticity, muscle weakness, atrophy, and fasciculations, which progressively lead to paralysis and ultimately respiratory failure [3]. While ALS has historically been considered a pure motor neuron disease (MND), converging clinical, neuroimaging, and neuropathological evidence now indicates that it is a multisystem disorder with widespread central nervous system (CNS) involvement beyond the motor pathways. Cognitive impairment, particularly affecting frontal and temporal functions, is observed in a significant subset of patients, highlighting non-motor cortical involvement. At the cellular level, disturbances in mitochondrial function, glutamate metabolism, and glial interactions further underscore its nature as a disorder of multiple biological processes, rather than one restricted solely to motor neurons [4].

The difficulty in diagnosing and managing ALS arises from its heterogeneity. This challenge arises from its heterogeneous clinical presentation, multiple disease phenotypes, and overlap of symptoms and signs with those of other neurological and neuromuscular disorders [5]. In the early diagnostic stages, patients presenting with progressive dysarthria, dysphagia, limb weakness, or unexplained respiratory failure should be promptly referred to a neurologist for further evaluation. Such recommendations are consistent with the guidance of patient advocacy groups, which emphasize early referral not only to improve timely treatment but also to increase opportunities for enrolment in clinical trials.

Importantly, ALS is increasingly recognized as part of a broader clinicopathological spectrum with frontotemporal dementia (FTD) [6,7]. Population-based phenotyping studies reveal that up to 50% of ALS patients exhibit measurable cognitive or behavioral impairment, and approximately 13% meet the diagnostic criteria for concomitant behavioral-variant of FTD [8,9]. This overlap underscores the shared molecular and pathological mechanisms linking the two conditions [10]. One of the most prominent pathological hallmarks uniting ALS and FTD is the abnormal cytoplasmic aggregation of TAR DNA-binding protein 43 (TDP-43) [11,12]. Under physiological conditions, TDP-43 is a predominantly nuclear protein involved in RNA splicing, transport, and regulation. In disease states, however, TDP-43 mislocalizes from the nucleus to the cytoplasm, where it undergoes abnormal phosphorylation, ubiquitination, and cleavage, ultimately forming insoluble inclusions. These aggregates are strongly implicated in neurotoxicity, synaptic dysfunction, and neuronal death. Consequently, ALS and FTD are now frequently classified as TDP-43 proteinopathies, reflecting the central role of this protein in disease pathogenesis [10,13].

ALS presents in several clinical variants that appear distinctive, raising the question of whether they represent separate disease entities with distinct biologies or represent ends of a continuum [14]. Neuropathological evidence largely supports the latter, suggesting that differences arise primarily from the anatomical distribution of the pathological burden rather than fundamental biological divergence. Alongside these variants are several genetic syndromes that affect the motor system but are considered distinct from ALS, although they may cause diagnostic confusion. The principal ALS variants include Primary Lateral Sclerosis (PLS), Progressive Muscular Atrophy (PMA), and overlap forms with FTD. At the same time, related conditions such as Spinal Muscular Atrophy (SMA) and Hereditary Spastic Paraparesis represent important differential diagnoses [15].

Beyond genetic predisposition, a variety of environmental and lifestyle factors have also been proposed to contribute to ALS pathogenesis. Reported associations include smoking, alcohol consumption, antioxidant intake, body mass index, physical exercise, head trauma, metabolic and inflammatory states, and occupational or environmental exposures to metals, pesticides, and electromagnetic fields [1,16]. Although age, gender, and family history are well-established risk factors, intense physical activity and frequent head injuries may increase the risk of ALS in people who are genetically predisposed [17]. Furthermore, geographical clustering of presymptomatic ALS cases supports the involvement of exogenous exposures in disease development, though the precise contribution of such factors remains incompletely understood [1].

In addition to genetic and cellular mechanisms, systemic factors have recently gained attention in ALS research, particularly the gut–brain axis. Emerging evidence suggests that alterations in gut microbiota composition may influence neuroinflammation, metabolic signaling, and immune responses, thereby contributing to motor neuron vulnerability. Although this area remains under active investigation, microbiome-related mechanisms may be important modifiers of disease progression and potential therapeutic targets. Therefore, understanding the interactions among ALS genetics, neurodegeneration, and the gut microbiota may provide new insights into disease pathogenesis and treatment strategies.

Among the non-genetic factors, a significant body of evidence has emerged suggesting that disturbances in the gut–brain axis, via alterations in gut microbiota composition, may influence neuroinflammation, metabolic signaling, and immune responses that contribute to motor neuron diseases. Delineating these mechanisms could provide insight into disease mechanisms and open the door to novel disease strategies [18,19].

## 2. Epidemiology

ALS was first clinically described in the 19th century, with Jean-Martin Charcot formally identifying the disease in 1869. However, earlier work by Charles Bell in 1824 had already introduced the concept of motor-dependent diseases [18]. The condition gained wider recognition in the early 20th century following the diagnosis and subsequent death of baseball player Lou Gehrig, after whom ALS became popularly known as “Lou Gehrig’s disease” [18]. ALS presents in both familial (fALS, 5–10% of cases) and sporadic (sALS, 90–95% of cases) forms [18,19]. The mean age of onset differs between subtypes: fALS typically occurs between ages 47 and 53, whereas sALS typically occurs between ages 58 and 63. However, juvenile and late-onset cases have also been reported [18]. Clinical presentation varies, with spinal onset (≈75%) most common, followed by bulbar onset (≈25%) and, rarely, respiratory onset (≈3%) [18]. Survival is generally limited to 2–5 years post-diagnosis, though 20% of patients live beyond 5 years, and a small proportion may survive beyond 2 decades [18].

Epidemiological studies indicate that ALS is a rare but globally distributed disease with regional and demographic variation. Incidence rates range from 0.5 to 2.6 per 100,000 person-years, with higher rates reported in populations of European descent than in Asian populations [2,19]. For instance, standardized global incidence has been estimated at 1.68 per 100,000 person-years, but varies from 0.73 in South Asia to 2.25 in Oceania [2]. In Western Europe, incidence reaches 2.4 per 100,000 person-years, while population-based registries report 3.8 per 100,000 in Stockholm and Scotland [19]. Such variation may reflect differences in methodology, diagnostic standards, population structure, and genetic predisposition [2,19].

Incidence consistently increases with age, peaking between 60 and 79 years [2]. Men are at greater risk than women, particularly for sporadic forms, with an overall male-to-female incidence ratio of approximately 1.35 [18,19,20]. While some studies report stable rates over recent decades, others suggest a gradual increase in both incidence and mortality, possibly due to improved case recognition and reporting [21]. Importantly, the prevalence of ALS is expected to rise in the future, driven by aging populations and improvements in disease management that extend survival [22]. Collectively, these findings highlight ALS as a complex neurodegenerative disorder shaped by an interplay of genetic, environmental, and demographic factors, reinforcing the need for well-curated population registries to generate accurate epidemiological data.

## 3. Proposed Disease Mechanisms

ALS is caused by a confluence of pathogenic processes that operate in tandem to create selective motor neuron vulnerability at the cellular and molecular levels. With indications of increased reactive oxygen species (ROS) generation and compromised antioxidant defenses, oxidative stress appears to be a significant contributing factor [23]. Axonal transport is further disrupted, and motor neuron degeneration is accelerated by oxidative damage to proteins such as neurofilaments. In contrast, mutant superoxide dismutase 1 (*SOD1*), linked to familial ALS, can catalyze the formation of aberrant free radicals [24]. This is closely related to glutamate-mediated excitotoxicity, in which a self-replicating cycle of neuronal damage is driven by impaired glutamate clearance, mostly due to malfunction of the glial transporter EAAT2. This results in ongoing calcium influx, mitochondrial overload, and additional ROS production [24].

An additional defining feature of ALS pathogenesis is mitochondrial dysfunction. Both patient tissue and animal models have shown structural abnormalities, including vacuolated and enlarged mitochondria, decreased calcium-buffering capacity, and reduced oxidative phosphorylation [24,25]. These mitochondrial abnormalities increase the vulnerability of metabolically demanding motor neurons to oxidative and excitotoxic stress and impair their energy supply [26]. Simultaneously, ALS is characterized by aberrant protein aggregation, including dipeptide repeat proteins (DPRs) from C9ORF72 expansions, ubiquitinated deposits, and Bunina bodies, which contribute to disturbed proteostasis [25]. The ALS-FTD spectrum is also defined by mislocalized and aggregated TDP-43, which is essential for RNA dysregulation and neuronal death [4].

Beyond neuron-intrinsic defects, non-neuronal cells also significantly influence ALS pathogenesis. Activated microglia and astrocytes release nitric oxide, cytokines, prostaglandins, and glutamate, amplifying both oxidative and excitotoxic damage, while Schwann cell dysfunction compromises axonal maintenance and regeneration [4,24]. These neuroinflammatory processes establish a toxic feedback loop that accelerates neuronal degeneration [27]. In addition, cytoskeletal and axonal transport defects further impair long-distance communication within motor neurons, compounding their vulnerability [24]. Ultimately, many of these processes converge on apoptotic cell death pathways, including activation of caspases, release of cytochrome c from mitochondria, and altered regulation of Bcl-2 family proteins, consistent with programmed cell death [28].

Taken together, ALS is best understood as a multifactorial neurodegenerative disorder in which oxidative stress, excitotoxicity, mitochondrial dysfunction, protein aggregation, axonal transport defects, and neuroinflammation act synergistically to drive motor neuron loss. Importantly, the interplay between intrinsic neuronal vulnerabilities and extrinsic glial responses underscores the disease’s heterogeneity and complexity and highlights why ALS is increasingly recognized as a multisystem disorder rather than a purely MND [4].

## 4. ALS Clinical Features

The clinical spectrum of ALS is remarkably heterogeneous, reflecting differences in the involvement of UMN and LMN, anatomical sites of onset, and disease progression patterns. Recognized MND phenotypes include classic ALS, bulbar, flail arm, flail leg, pyramidal and respiratory variants, as well as PLS and PMA. PLS is defined predominantly by pure UMN degeneration, whereas PMA is characterized by LMN involvement [1,2]. Importantly, recent genetic studies demonstrate that ALS-causative genes are not uniquely associated with a single clinical form but may contribute to a wide spectrum of phenotypes, ranging from predominant LMN to UMN manifestations [1].

Bulbar ALS is among the homogeneous phenotypes, with relatively consistent clinical and pathological features. It is typically associated with rapid progression, short survival (<2 years post-diagnosis), and significantly reduced quality of life [1,2]. Pseudobulbar palsy represents another bulbar-dominant variant, in which corticobulbar tract dysfunction leads to dysarthria, dysphagia, and limb involvement over time. Females are more frequently affected than men, and survival is longer than in classic bulbar ALS. Patients often experience pseudobulbar affect, a syndrome of involuntary emotional expression, which has been linked to cortico-pontine–cerebellar circuits and may be present in up to 50% of ALS and PLS patients [1]. A rarer bulbar phenotype is facial onset sensory motor neuropathy (FOSMN), which begins with trigeminal sensory impairment, later progressing to orofacial weakness, dysphagia, limb involvement, and cognitive-behavioral impairment of a frontotemporal type. Pathological evidence suggests that FOSMN belongs to the group of TDP-43 proteinopathies [29,30].

Respiratory-onset of ALS, though rare (≈3–5% of cases), is clinically significant because it is often misdiagnosed until patients present with respiratory failure or require intubation. It predominantly affects older men, often with LMN features, weight loss, and rapid progression. Early recognition is crucial for timely initiation of non-invasive ventilation [1].

From a broader perspective, ALS manifests as combined UMN and LMN dysfunction across bulbar, cervical, thoracic, and lumbar segments. This results in progressive weakness of voluntary skeletal muscles responsible for speech, swallowing, limb movements, and respiration, with different clinical presentations [2,31]. Bulbar- and spinal-onset phenotypes each account for about one-quarter to one-third of cases. At the same time, flail arm and flail leg variants, respiratory onset, hemiplegic ALS, PLS, and PMA occur less frequently [32].

Classic ALS is most often spinal-onset, presenting around the age of 60 with asymmetric, painless limb weakness. Clinical examination reveals fasciculations, muscle atrophy, and weakness consistent with LMN involvement, combined with hyperreflexia and spasticity, indicating UMN dysfunction. Bulbar-onset ALS, present in ~20% of patients, manifests with dysarthria, dysphagia, tongue fasciculations, brisk jaw jerk, and often pseudobulbar affect. Aspiration, poor nutrition, and early respiratory failure are the main causes of the negative diagnosis, leading to a median survival of about two years [32]. Respiratory-onset ALS, though uncommon (~3–5%), has the worst prognosis, with a mean survival of ~1.4 years [32].

Population-based studies further refine ALS classification into eight recognized phenotypes: classic, bulbar, flail arm, flail leg, pyramidal, respiratory, pure LMN, and pure UMN. Each phenotype is distinguished by onset site, progression pattern, and neurophysiological features. For instance, flail arm ALS is defined by proximal upper-limb weakness, while flail leg ALS is characterized by distal lower-limb wasting and weakness. Pyramidal ALS shows severe spasticity with Babinski or Hoffmann signs, while PLMN and PUMN reflect more isolated neuronal system involvement. These categories underscore ALS’s marked heterogeneity and the need for precise classification to guide prognosis and clinical management [33].

Together, these findings emphasize that ALS is not a single, uniform disorder but a spectrum of overlapping phenotypes shaped by genetic, demographic, and clinical factors. Recognizing this heterogeneity is essential for diagnosis, prediction, and the creation of customized treatment plans.

## 5. Diagnosis of ALS

The diagnosis of ALS remains primarily clinical, supported by neurophysiological testing and guided by standardized diagnostic criteria. Patients most frequently exhibit increasing weakness, which can affect any part of the body but usually begins in the terminal limbs, including the bulbar, cervical, and thoracic regions [34]. The indicator of ALS diagnosis is the combination of UMN and LMN signs across multiple body regions. LMN features include weakness, atrophy, fasciculations, and hyporeflexia, while UMN signs include spasticity, hyperreflexia, and slowed voluntary movement. In bulbar involvement, clinical findings may include dysarthria, dysphagia, poor palatal elevation, and tongue atrophy or fasciculations. Respiratory abnormalities may also emerge, often presenting as a weak or soft voice and reliance on accessory respiratory muscles [35].

Electrophysiological testing, including nerve conduction studies (NCS) and electromyography (EMG), is critical for excluding alternative diagnoses and improving diagnostic sensitivity [36]. In ALS, the NCS results are typically normal or mildly abnormal, with reduced compound motor action potential amplitudes consistent with axonal loss, but without conduction block [36]. EMG reveals evidence of widespread denervation across proximal and distal muscles in multiple segments, supporting a diagnosis of ALS when clinical evidence is not yet fully obvious [34].

The diagnostic criterion has historically been set by the World Federation of Neurology’s Revised El Escorial (rEE) Criteria, which require evidence of LMN injury, UMN involvement, progressive symptom spread, and exclusion of other possible causes. However, their strictness frequently prevents individuals with early disease from being included in clinical trials and delays diagnosis, which hinders timely care [34,37].

The Gold Coast (GC) diagnostic criteria are intended for both clinical use and trial inclusion, whereas the revised El Escorial criteria were developed for clinical trials (Table 1). It was found that the GC criteria are appropriate for trial enrolment [38]. This opens the door to initiating disease-modifying therapies earlier in patients with isolated LMN involvement, but not in those with isolated UMN signs [39]. According to these condensed recommendations, ALS is defined as progressive motor impairment, at least one body region’s UMN and LMN dysfunction, and the exclusion of other diagnoses. Unlike the Revised El Escorial framework, the GC Criteria remove the categories of “possible,” “probable,” and “definite” ALS that were used in the El Escorial criteria, thereby reducing diagnostic uncertainty. Research shows that these criteria allow for an earlier diagnosis while maintaining high sensitivity and specificity, especially in patients with atypical or slowly advancing forms of ALS [37].

The average diagnostic delay, reflecting both systemic and clinical obstacles, remains between 10 and 16 months, despite improvements in diagnostic criteria [40]. It is recommended that the Gold Coast Criteria be widely incorporated into standard clinical practice to improve patient suitability for clinical trials, enhance early detection, and provide rapid access to broad-ranging treatment [38,40].

## 6. Pathophysiology

The pathophysiology of ALS is complex and not fully understood. Current understanding indicates that the disease arises from a combination of genetic mutations and cellular abnormalities that affect common neuropathological pathways, including RNA metabolism, protein recycling, mitochondrial dysfunction, oxidative stress, cytoskeletal formation, and DNA repair [41,42,43,44,45,46,47,48,49,50]. Genomic studies show that ALS is only partly explained by known mutations, with over 40 genes linked to disease risk. The most common and clinically significant among them are C9orf72, TARDBP (which encodes TDP-43), FUS, and SOD1. At the cellular level, disturbances in the protein degradation systems represent a critical pathological axis. Mammalian cells primarily use the ubiquitin-proteasome system to degrade small, soluble proteins. They use autophagy to remove larger protein aggregates. In ALS, problems in both systems lead to the buildup of insoluble inclusions, especially TDP-43. This misplacement and cytoplasmic clumping are almost always present, observed in about 97% of ALS cases [46,48]. This accumulation further impairs RNA splicing, including of key neuronal transcripts such as stathmin-2 (STMN2), which is essential for axonal growth and motor neuron function [51].

Mitochondrial dysfunction is another key feature of ALS development. Post-mortem studies of sporadic ALS cases have shown reduced activity of electron transport chain complexes I, II, III, and IV in the spinal cord. They also found decreased ATP production in peripheral lymphocytes and structural problems in neuronal mitochondria [52]. Mutant *SOD1* further inhibits the voltage-dependent anion channel, exacerbating bioenergetic failure and promoting apoptosis [51]. Oxidative stress is closely linked to these mitochondrial problems. Increased levels of ROS and reactive nitrogen species, along with reduced antioxidant defenses such as glutathione, make motor neurons more vulnerable [47]. Dysregulation of the Nrf2-mediated antioxidant response further aggravates oxidative injury, highlighting the therapeutic potential of targeting redox homeostasis [47].

Overall, the pathophysiology of ALS is best understood as the result of multiple genetic and molecular factors that harm neuronal survival in various ways. Instead of being caused by a single pathway, ALS is a multisystem disorder. Impaired RNA metabolism, protein handling failures, mitochondrial dysfunction, oxidative stress, and neuroinflammation interact in complex ways to drive the onset and progression of the disease. These insights emphasize the importance of treatment approaches that focus not only on specific molecular issues but also on the broader network of pathways that contribute to neurodegeneration in ALS.

## 7. Introduction to the Gut Microbiome

The disruptions in gut function and microbiota dysbiosis have been documented in various chronic and neurodegenerative disorders, including Alzheimer’s disease, Parkinson’s disease, and multiple sclerosis [53,54]. The gut–brain–microbiota axis is thought to be a potential modifier of ALS progression, representing a bidirectional communication system linking the CNS and the gastrointestinal tract through neuronal, immune, and metabolic pathways [55,56]. Frequently experiencing gastrointestinal symptoms and dysphagia may correlate with neurodegenerative disease severity and progression, including constipation, abnormal bowel motions, and abdominal pain [53,57]. Growing evidence indicates correcting gut dysbiosis with dietary treatments, probiotics, or other microbiome-modulating techniques may help reduce symptoms and mimic disease development [54,58].

Studies conducted on *SOD1* transgenic mice, which account for approximately 10% of familial ALS cases due to *SOD1* mutations, revealed changes in the butyrate-producing gut microbiota and worsened disease progression by driving microglial transition to a neurodegenerative phenotype [59,60,61]. Microbiome modifications increase Lipopolysaccharide (LPS) levels, which activate Toll-like receptor 4 (TLR4) on astrocytes and microglia, activating neuroinflammatory pathways linked to ALS progression [62]. Researchers genetically modified mice to acknowledge the pathophysiology and the development of the disease. The characteristics of ALS can be studied using HSOD1G93A mouse models, which carry the human *SOD1* gene with the G93A point mutation [63]. It was observed that a significant rise in oxidative stress markers, inflammatory cytokines, and apoptotic regulators promoted disease progression. Notably, fibrosis-related proteins were upregulated even at the pre-symptomatic stage. These findings support a molecular relationship among oxidative stress, hepatic inflammation, and the development of liver fibrosis in ALS mouse models [64].

### 7.1. Significant Bacterial Taxa in ALS-Associated Dysbiosis

**Figure 1:**
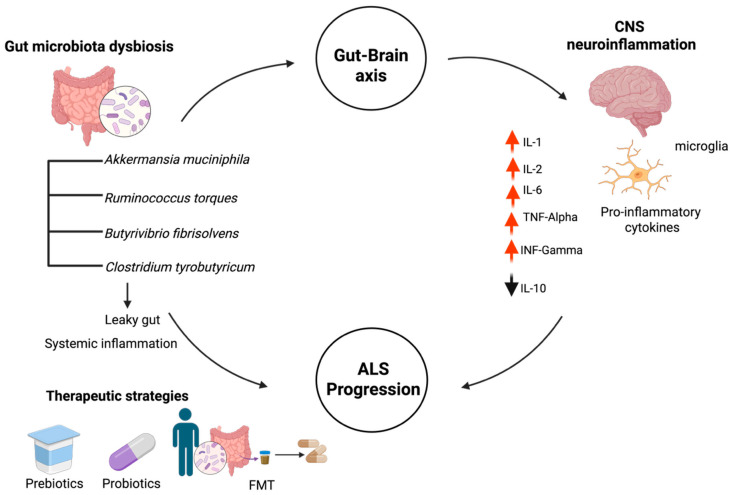
Represent the link between gut microbiota imbalance and ALS progression through the gut–brain axis. The pathway highlights how a decrease in beneficial bacteria and the resulting ‘leaky gut’ lead to systemic and neuroinflammatory responses driven by microglia. The imbalance in cytokines, marked by increased pro-inflammatory markers (TNF-α, IFN-γ) and reduced anti-inflammatory signaling (IL-10), creates a cycle that worsens motor neuron degeneration. The figure also highlights prebiotics, probiotics, and FMT as potential treatment options to modulate this axis and reduce disease severity. Generated through biorender http://www.biorender.com/.

Several bacterial taxa have been linked to an altered gut microbiome in individuals with ALS, with variable frequencies and functional importance. While past studies [63,65] have focused on alterations in common genera such as *Bacteroides*, *Prevotella*, and *Lactobacillus*, new data highlight the importance of specific species with pronounced metabolic effects on gut–brain communication. Among the microbiota, four species are well known to influence ALS disease progression: *Akkermansia muciniphila*, *Ruminococcus torques*, *Butyrivibrio fibrisolvens*, and *Clostridium tyrobutyricum* [58,65,66]. These microbiotas play critical roles in mucin degradation, short-chain fatty acid (SCFA) synthesis, and the regulation of the neuroinflammatory pathway in ALS, as illustrated in Figure 1. The following discussion covers their known changes in ALS and possible connections to its progression.

Commencing with the most promising probiotic, *Akkermansia muciniphila,* a gut bacterium that occupies the gut mucosa, was first discovered in 2004 [67]. It has a role in maintaining gut barrier function and integrity by degrading mucin (a protective layer in the intestines) [68]. Keeping toxic compounds out of the bloodstream while also controlling inflammation [69]. Using metagenomic and metabolomic techniques, researchers have identified a link between gut microbiota and the severity of ALS. In particular, they discovered that a microbiome rich in *Akkermansia muciniphila* improved symptoms in SOD1-Tg ALS mice [70]. In contrast, *Ruminococcus torques* and *Parabacteroides distasonis* exacerbated ALS progression [66]. *R. Torques* might lead to dysbiosis and decrease nicotinamide biosynthesis, while *P. distasonis* alters systemic metabolite profiles, which influence NAD^+^ balance and mitochondrial function [58,71].

Supplementation with *A. muciniphila* increased nicotinamide levels in the nervous system, a neuroprotective metabolite that has been demonstrated to improve motor performance and spinal cord gene expression [56]. Both therapies boosted neuroprotective genes related to mitochondrial integrity, NAD^+^ homeostasis, and oxidative stress reduction, which are impaired in ALS. To determine their clinical significance, researchers analyzed the gut microbiomes of ALS patients and healthy family members [72]. Notably, ALS patients had significantly lower levels of nicotinamide in serum and cerebrospinal fluid [72,73]. These findings are consistent with previous studies in ALS animal models and indicate that microbiome-associated nicotinamide deficiency may contribute to disease development. This human evidence supports the possibility of microbiome-targeted therapeutics and establishes the framework for future clinical trials.

*Butyrivibrio fibrisolvens* and *Clostridium tyrobutyricum* ferment dietary fibers and undigested carbohydrates primarily in the colon under anaerobic conditions, producing SCFAs, notably butyrate [65,74]. Butyrate plays a critical role in host energy metabolism, intestinal barrier integrity, and neuroimmune signaling [75]. Butyrate activates G protein-coupled receptors (GPCRs) and inhibits histone deacetylases, altering gene expression, decreasing inflammation, and promoting brain health [75]. Evidence from transgenic G93A ALS mouse models indicates that *B. fibrisolvens* is significantly reduced even before clinical symptom onset, suggesting that gut dysbiosis is an early contributor to disease progression. This microbial imbalance reduces beneficial SCFAs while increasing harmful metabolites such as LPS, which promote systemic and neuronal inflammation [65].

In parallel, experimental colonization of germ-free mice with *C. tyrobutyricum* indicates its ability to restore SCFA balance, strengthen gut barrier function, and alter the enteric neuroimmune system, all of which are highly significant in ALS pathogenesis [76]. Overall, the reduction in butyrate-producing species such as *B. fibrisolvens* and *C. tyrobutyricum* may represent a crucial factor linking gut dysbiosis to ALS progression, and their restoration through probiotics, dietary modulation, or microbiota-targeted interventions has emerged as a promising therapeutic strategy [77].

### 7.2. Cytokines as ALS Biomarkers

Cytokine profiling has emerged as a promising biomarker strategy in ALS, providing insight into the neuroinflammatory landscape and its association with disease progression. Classical mediators such as IL-6, IL-1β, and TNF-α are among the most consistently elevated in serum, cerebrospinal fluid (CSF), and spinal cord tissue samples from ALS patients [78]. Such cytokines would also be potential biomarkers of disease progression, driving astrocyte activation, inflammasome signaling and excitotoxic motor neuron death [79,80]. Beyond these classical markers, alterations in IL-2, IL-10 and IFN-γ also hold diagnostic and prognostic ic value [81,82]. Elevated serum IL-2 is associated with aberrant homeostasis of regulatory T cells (Treg), and low-dose IL-2 therapy has been shown to expand Regulatory T Cell populations and delay symptom onset in a subset of patients [80]. IL-10, a key anti-inflammatory cytokine, is often increased in patient biofluids, with higher levels linked to slower disease progression and extended survival in ALS mouse models [83,84]. Conversely, IFN-γ is typically elevated in serum and CSF, often as a downstream effect of IL-18 signaling. While it is often associated with faster progression, it may also have neuroprotective effects, depending on dose and duration [85,86]. These cytokines, when considered collectively, serve not only as fluid indicators but also as molecular drivers of neurodegeneration, with their quantitative and diagnostic relevance summarized in Table 2, and their broader role in ALS-associated neuroinflammatory networks illustrated in Figure 1.

The microglia, also known as the resident, innate immune cells of the CNS, adopt a disease-associated phenotype in ALS characterized by chronic neuroinflammation driven by NF-κB and NLRP3 inflammasome activation [87,88]. Activated microglia secrete persistently elevated TNF-α and IL-6, perpetuating a neurotoxic milieu that injures motor neurons [78]. Concurrent NLRP3 signaling processes and releases IL-1β, further amplifying local inflammation [89]. In G93A-SOD1 mice, transcriptomic and proteomic analyses reveal sustained upregulation of TNF-α and IL-6 in symptomatic microglia [90]. This pro-inflammatory cytokine storm promotes astrocyte activation and peripheral immune cell infiltration, exacerbating neuronal degeneration. Pharmacological or genetic inhibition of microglial NF-κB reduces IL-1β, IL-6, and TNF-α secretion, delays disease onset, and highlights a promising therapeutic avenue [91]. 

### 7.3. FMT Therapy

Interesting outcomes have come from recent clinical studies following fecal microbiota transplantation (FMT) in ALS patients (Figure 1) [92,93,94]. While FMT recipients reported improvements in constipation, depression, and anxiety, as well as a sustained increase in Bifidobacterium abundance, a 2022–2023 double-blind randomized trial in China with 27 sporadic ALS patients revealed no significant difference in ALS Functional Rating Scale-Revised (ALSFRS-R) scores between FMT and placebo groups over 35 weeks [93].

Currently, 42 ALS patients are undergoing digestive tract infusions at baseline and at 6 months as part of another ongoing European trial (NCT03766321) that is assessing the immunomodulatory effects of FMT, specifically its effects on regulatory T cells; outcomes include changes in the microbiome, respiratory function, and disease progression [95]. Furthermore, preclinical research in ALS animal models showed that modulating the gut microbiota delayed disease progression, and ALS Untangled reported a case series in which two patients showed clinical improvement following FMT [96].

Post-FMT metagenomic and metabolomic analyses showed a significant increase in beneficial gut microbial species, particularly *Bacteroides* (*Bacteroides stercoris*, *Bacteroides uniformis*, *Bacteroides vulgatus*) and *Faecalibacterium prausnitzii*, indicating a shift toward a more neuroprotective, anti-inflammatory microbial composition. Microbial composition. Despite the study’s encouraging findings, the limited number of participants and the short follow-up period (4–6 months) highlight the need for larger, longer-term clinical trials to demonstrate that FMT is a suitable alternative treatment for ALS with respiratory failure [94]. 

### 7.4. Probiotics

Early ideas about “beneficial microbes” from Nobel laureate Elie Metchnikoff have evolved into the current definition of probiotics. Probiotics are live microorganisms that, when given in sufficient amounts, provide health benefits to the host [97]. In addition to their local effects on gut balance, probiotics affect the composition and structure of the gut microbiota. This, in turn, strengthens the intestinal barrier, reduces the growth of harmful bacteria, and regulates the body’s immune response. Evidence suggests that problems with the gut–brain axis may contribute to disease progression [58,98].

The metabolites of gut bacteria, especially SCFAs, play key roles as signaling molecules in the gut–brain axis. SCFAs influence immune responses and act as neuroactive compounds that affect neuronal health and CNS function. Experimental models show that probiotics, which can increase SCFA-producing microorganisms like oxalate-producing bacteria, can restore microbial balance, normalize intestinal antimicrobial peptide expression, and extend survival in ALS mice by more than a month [97]. Similarly, bacterial species such as *Akkermansia muciniphila* and *Faecalibacterium prausnitzii* have been shown to have positive effects. They promote mucin breakdown, improve intestinal barrier function, and raise nicotinamide levels in the CNS of ALS-prone SOD1G93A mice [98].

Specific bacterial groups and probiotic mixtures are being studied for their potential therapeutic effects. *Bifidobacterium* and *Lactobacillus* strains, known for their probiotic benefits, have been shown to influence inflammation, cognitive performance, and blood–brain barrier integrity in animal models of neurodegeneration [58]. Interestingly, studies with Caenorhabditis elegans containing ALS-related proteins (FUS and TDP-43) found that many probiotic blends provided minimal benefit. In contrast, *Lacticaseibacillus rhamnosus* HA-114 significantly slowed neurodegeneration and paralysis, suggesting that some strains may be more effective for ALS-related issues [58].

These studies suggest that changing the gut microbiota could offer valuable treatment options for ALS (Figure 1). Although the evidence is still growing, it suggests that probiotic supplements and microbial metabolites might slow disease progression by modulating the immune system, protecting nerves, and improving communication between the gut and the CNS.

### 7.5. Prebiotics

Prebiotics are non-digestible dietary components that selectively change the composition and activity of the gut microbiota (Figure 1). They are increasingly recognized as potential factors in the gut–brain axis in ALS [98,99]. Prebiotics support the production of SCFAs such as butyrate, acetate, and propionate [98]. These microbial byproducts are important for maintaining gut barrier integrity, regulating immune responses, and supporting overall body balance. In the context of ALS, prebiotics may help counter the lower SCFA levels often seen in patients. This can improve both intestinal function and blood–brain barrier function, which are frequently affected in neurodegenerative diseases [71].

A variety of naturally occurring prebiotics can be found in common foods like fruits, vegetables, cereals, and edible plants. Examples include inulin, oligofructose, galactooligosaccharides (GOS), pectin, and resistant starch. These compounds are easily fermented by gut bacteria [98,99]. In addition, some synthetic compounds, such as lactulose, fructooligosaccharides (FOS), maltooligosaccharides, and cyclodextrins, have been developed to mimic or enhance prebiotic effects. They offer controlled ways to influence the gut microbial community [98]. Together, these compounds serve as food for beneficial bacteria, helping shape the microbial environment and potentially providing overall benefits for ALS patients.

Polyphenols are an important group of prebiotics associated with ALS. These plant-based compounds include quercetin, catechins, resveratrol, curcumin, and pterostilbene. They are studied for their antioxidant, anti-inflammatory, and neuroprotective effects [65,99]. Research suggests that polyphenols can regulate immune responses, remove ROS, and change gut microbiota composition. These effects may help reduce the neuroinflammatory response seen in ALS.

Preclinical studies further support the potential of prebiotics in ALS. In SOD1-G93A transgenic mouse models, adding GOS-rich yogurt delayed disease onset. It also improved mitochondrial function, reduced neuroinflammation, and extended overall lifespan [100]. Likewise, using FOS alone or in combination with GOS boosted the growth of Bifidobacterium species. It also affected signaling pathways shared by colonic and cortical tissues, suggesting a direct effect of gut changes on CNS issues [100]. Notably, these benefits were accompanied by reductions in muscle loss and atrophy. This shows that the positive effects of prebiotics go beyond the gut and into neuromuscular health.

### 7.6. Genomics of ALS

**Figure 2:**
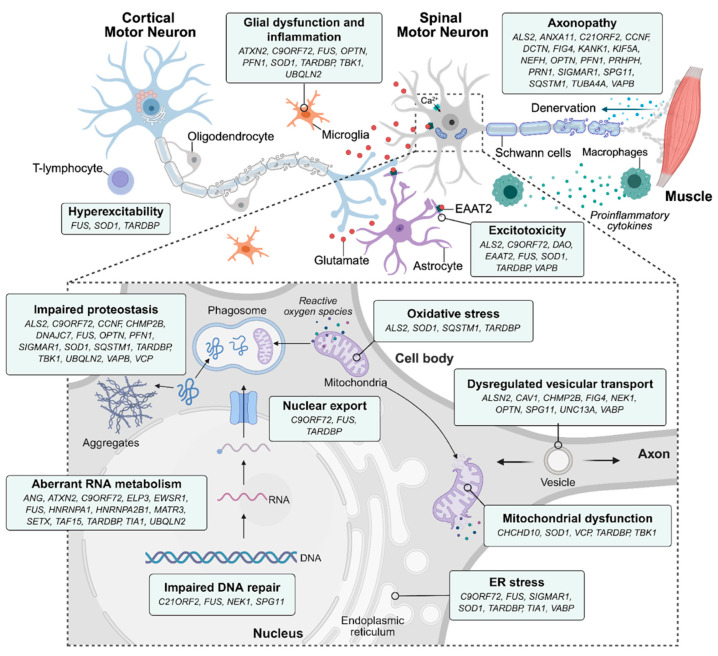
Genes implicated in ALS pathogenesis are grouped by the underlying cellular and molecular processes. Repurposed from Verdés, Navarro and Bosch (2025) with permission [104].

Genetic discoveries have reshaped the understanding of motor neuron disorders, shifting research focus toward molecular mechanisms and targeted gene-based therapies. Although around 90% of ALS cases occur sporadically, around 10-15 percent of ALS cases are familial and are caused by inherited mutations [101]. Over the past decades, several studies have revealed that the development of the disease involves several mutated genes, including *C9orf72*, *SOD1*, *TARDBP*, and *FUS* [102] (Figure 2) Genetics discoveries of ALS have not only enhanced our understanding of the disease mechanisms but also provided a chance to develop a specific treatment, with gene therapy emerging as a promising and potentially revolutionary approach [103,104]. This section summarizes recent findings on the genes implicated in ALS and examines the effectiveness of gene therapy, addressing their clinical implications, current therapeutic approaches, ongoing trials, and prospects.

#### 7.6.1. C9orf72

The most common genetic cause of familial ALS is a hexanucleotide (GGGGCC) repeat expansion of the C9orf72 gene [105]. First identified in 2011, it has since been recognized as the major genetic contributor to ALS worldwide. However, its frequency varies substantially across populations and ethnic groups. For example, Edgar et al. (2021) reported in a Malaysian cohort that the repeat expansion was present in only a subset of patients, reflecting this geographic and ethnic heterogeneity [102].

The effects of the C9orf72 mutation have been widely studied, with consistent evidence across Chinese and European cohorts showing that carriers are more likely to present with bulbar or early-onset ALS [106,107]. These cases are often marked by speech and swallowing difficulties, rapid functional decline, and overall reduced survival rate, highlighting the aggressive clinical course associated with pathogenic repeat expansion [107]. Collectively, findings suggest that C9orf72 represents a universal risk and severity factor in ALS, although its prevalence varies geographically.

Three main proposed mechanisms explain the pathogenicity of C9orf72 mutations. First, a gain-of-function phenotype occurs when repeat RNA sequesters RNA-binding proteins essential for normal function. Second, repeat-associated non-ATG (RAN) translation of the GGGGCC sequence generates toxic DPRs, which accumulate in neurons. Third, a loss-of-function (LOF) mechanism may result from reduced C9orf72 protein levels. The interplay of these three mechanisms is thought to drive C9orf72-related ALS pathogenesis [108]. To conclude, understanding these processes is crucial, as C9orf72 not only informs the disease mechanisms underlying both monogenic and sporadic ALS but also highlights the need to develop targeted therapeutic strategies for C9orf72-related ALS [108].

#### 7.6.2. SOD1

Mutations in superoxide dismutase 1 (SOD1) were the first genetic defects identified in ALS, discovered in the early nineties. The SOD1 gene is located on chromosome 21q22.11 and encodes a 153-amino-acid monomeric protein [109]. It is highly conserved throughout evolution, ubiquitously expressed, and accounts for 1–2% of total soluble protein in the CNS. The translated SOD1 protein is a crucial enzyme that acts as a radical antioxidant defense mechanism against oxidative stress in cells, helping maintain cellular homeostasis [110].

The most frequent variants of the SOD1 gene, such as D90A, A4V, and G93A, are associated with both gain and LOF effects. These deleterious mutations alter SOD1 enzymatic activity, leading to the accumulation of highly toxic hydroxyl radicals. The resulting oxidative stress contributes to the degradation of nuclear and mitochondrial DNA and to protein misfolding, a hallmark of ALS pathology [111]. Gain-of-function toxicity involves aggregation and destabilization of dimers, leading to insoluble mutant SOD1 aggregates that disrupt cellular homeostasis and exert neurotoxic effects [112].

Clinical studies have further underscored the pathogenic role of SOD1 mutations. For example, Edgar et al. (2021) identified multiple pathogenic SOD1 variants associated with distinct clinical outcomes [102]. Jiang et al. (2024) reported that carriers of SOD1 mutations developed ALS symptoms several years earlier than noncarriers in China, with significantly higher mortality, confirming the gene’s role as both a disease driver and a factor accelerating progression [106]. These results are consistent with those of McFarlane et al. (2025), who reported identical trends across European ALS populations. They observed that SOD1 carriers typically present with spinal-onset disease, beginning with initial limb weakness, followed by rapid progression to respiratory failure and death [107].

Collectively, these findings demonstrate that SOD1 mutations are among the earliest and strongest genetic determinants of ALS severity and provide critical insight into disease mechanisms. Thus, SOD1 remains a central focus in both clinical research and therapeutic development, particularly in emerging gene therapy trials. 

#### 7.6.3. TARDBP

The *TARDBP* gene encodes the DNA-binding protein 43 (TDP-43), which plays a vital role in RNA processing, including splicing, transport, and stability [113]. Pathogenic TARDBP mutations result in abnormal cytoplasmic accumulation of TDP-43 aggregates, a hallmark of ALS pathology observed not only in mutation carriers but also in most sporadic ALS cases.

Mutations in TARDBP account for approximately 3–5% of familial ALS cases and less than 1% of apparently idiopathic cases. To date, more than 80 ALS-associated dominant mutations have been described, most of which affect the C-terminal domain of TDP-43 [114]. Mutations can also occur in the N-terminal domain, which is critical for TDP-43 self-association into oligomers necessary for RNA processing. While most TARDBP mutations are missense variants, rarer forms include truncating mutations (e.g., Tyr374Ter, Trp385IlefsTer10) and in-frame indels (e.g., Ser387delinsThrAsnPro). These alterations are associated with distinct ALS phenotypes and fibroblasts from patients carrying such mutations often express truncated TDP-43 protein isoforms supporting the functional impact of these variants [114].

Clinically, TARDBP mutations show variable frequencies and phenotypic heterogeneity across populations. In Malaysian cohorts, they were detected at relatively low frequency compared to C9orf72 and SOD1 [102]. In Chinese patients, TARDBP mutations were associated with diverse motor presentations and variable penetrance, highlighting significant clinical heterogeneity [106]. In European cohorts, carriers developed symptoms at younger ages compared to noncarriers, though disease progression varied widely, with some cases showing rapid decline and others slower progression [107].

Taken together, these findings underscore that while TARDBP mutations are less frequent, they remain clinically and pathogenically significant. Their contribution to ALS lies not only in their genetic impact but also in the near-universal presence of TDP-43 aggregates in ALS pathology, making TARDBP an intermediate-prevalence but high-impact gene in the disease’s genetic landscape.

#### 7.6.4. FUS

The FUS gene encodes a multifunctional protein involved in RNA metabolism, transcription regulation, and DNA repair [115]. Mutations in FUS, mostly heterozygous missense variants, account for approximately 2–4% of fALS and less than 1% [115]. FUS pathogenic mutants cause misallocation of FUS protein in the cytoplasm and form toxic aggregates that destroy motor neurons. Pathogenic mutations—particularly those clustering in the C-terminal region containing the nuclear localization signal- disrupt nuclear import, resulting in cytoplasmic mislocalization and aggregation of FUS and a corresponding loss of nuclear function, eventually leading to motor neuron loss.

FUS gene mutations are globally rare but have a strong clinical impact in ALS. They are often associated with very early onset and rapid disease progression [102,116]. Although uncommon among Southeast Asian populations, they are linked to severe and aggressive forms of the disease in both Chinese and European cohorts [107]. Collectively, these findings suggest that FUS, despite its rarity, plays a critical role in the most severe and early-onset subtypes of ALS.

Beyond the common *SOD1*, *C9orf72*, and *TARDBP* genes, several additional genes have been identified that play key roles in ALS pathogenesis, including *UBQLN2, TBK1, SQSTM1, OPTN, ATXN2*, and *NEK1*. These genes converge on pathways critical for maintaining cellular homeostasis, particularly the ubiquitin–proteasome system, autophagy, and neuroinflammatory regulation [117]. Disruptions in their function can lead to impaired protein degradation, accumulation of toxic protein aggregates, defective clearance mechanisms, and heightened inflammatory responses, ultimately contributing to the progressive degeneration of motor neurons characteristic of ALS [118].

#### 7.6.5. UBQLN2

Ubiquilin-2 (UBQLN2) protein is central to the proteasome and autophagy pathways, acting as a transporter that delivers ubiquitinated substrates to degradation machinery [119]. Ubiquitin, a highly conserved 76-amino-acid polypeptide expressed in all cell types, serves as a molecular tag that marks misfolded or damaged proteins for removal via a tightly regulated enzymatic cascade [120]. Disruption of this ubiquitin-dependent clearance process, caused by mutations in *UBQLN2*, leads to the accumulation of cytoplasmic protein aggregates and impaired neuronal homeostasis [121]. These pathogenic inclusions commonly contain TDP-43, SOD1, and FUS proteins, linking the disruption of ubiquitin-mediated clearance systems to ALS pathology [118].

Clinically, carriers often present with idiopathic or early-onset ALS accompanied by cognitive impairments [106]. Unlike most ALS-associated genes, UBQLN2 is X-linked, underscoring a potential sex-specific susceptibility to the disease.

#### 7.6.6. TBK1

One of the major ALS-associated genes is TANK-binding kinase 1 (TBK1), a highly conserved serine/threonine kinase that integrates multiple pathways of innate immunity, autophagy, and programmed cell death, making it vital for neuronal and organismal survival [122]. TBK1 promotes type I interferon signaling via IRF3 downstream of STING and RIG-I, regulates selective autophagy through phosphorylation of adaptors such as p62/SQSTM1, OPTN, NDP52, and suppresses RIPK1-driven necroptosis and apoptosis through direct phosphorylation [123].

Clinically, heterozygous LOF mutations in *TBK1* lead to haploinsufficiency and are strongly associated with ALS and FTD, accounting for 1–1.8% of ALS cases and up to 4% of familial ALS/FTD, while complete loss is embryonically lethal in animal models. Functional studies in zebrafish show that TBK1 knockdown or knockout causes motor neuron loss, impaired swimming, NAD^+^ depletion, and activation of necroptotic and apoptotic pathways, faithfully recapitulating ALS-like phenotypes [123].

Multi-omic analyses reveal the accumulation of neurotoxic metabolites such as quinolinic acid and proteomic upregulation of caspase-8 and RIPK1 orthologs, emphasizing TBK1’s role in metabolism and cell death regulation [123]. Therapeutically, treatment with the NAD^+^ precursor nicotinamide riboside restored motor behavior, while inhibition of necroptosis with necro-sulfonamide improved survival. Together, these findings establish *TBK1* as a multifunctional kinase whose dysfunction drives ALS pathology by disrupting autophagy, metabolic homeostasis, and regulated cell death [123].

#### 7.6.7. OPTN

*OPTN* (optineurin) is a multifunctional adaptor protein that plays pivotal roles in selective autophagy, including mitophagy, aggrephagy, and xenophagy, as well as vesicular trafficking and immune responses [124]. *OPTN* helps mediate cellular homeostasis not only by promoting autophagic degradation of damaged components, such as misfolded proteins or dysfunctional organelles, but also by interacting with signaling pathways that regulate cell survival and stress responses. Under conditions of cellular stress, *OPTN* may support cytoprotection by coordinating autophagy with inflammatory and apoptotic signaling, thereby preventing excessive damage. Dysregulation of *OPTN* function, whether through genetic mutation or altered regulation, can impair these protective mechanisms and potentially contribute to neurodegeneration and other pathologies [125].

Clinically, recent cohort analyses have demonstrated considerable genotype–phenotype heterogeneity among ALS patients carrying *OPTN* mutations [126]. In a large Chinese ALS cohort, 24 rare *OPTN* variants -including 17 novel mutations -were identified, and patients harboring pathogenic or likely pathogenic variants exhibited faster disease progression and shorter survival than those with benign variants. Moreover, the frequency of *OPTN* variants was higher in Asian ALS populations (1.08%) than in Caucasian ones (0.55%), emphasizing population-specific genetic contributions [126].

Collectively, these findings highlight *OPTN* as a critical regulator of neuronal homeostasis, whose dysfunction links impaired autophagy and inflammatory signaling with ALS pathogenesis, providing new insights into disease variability and potential therapeutic targets.

#### 7.6.8. SQSTM1

*SQSTM* (Sequestosome 1) encodes the multifunctional adaptor protein p62, which plays a central role in autophagy, proteostasis, and oxidative stress responses [127]. Mutations in *SQSTM1* disrupt the selective clearance of ubiquitinated protein aggregates, leading to accumulation of TDP-43 pathology, a key pathological hallmark of ALS [128]. Evidence from recent cohorts indicates that *SQSTM1* variants act as disease modifiers, with carriers exhibiting faster respiratory decline and more severe progression [129]. Mechanistically, p62 binds ubiquitin-tagged cargo and links it to LC3 on autophagosomes, thereby coordinating the degradation of misfolded proteins and damaged organelles. In addition, p62 modulates the Keap1-dependent activation of the Nrf2 pathway, inducing antioxidant gene expression under redox stress [130]. Collectively, *SQSTM1*/p62 functions as a central node linking autophagy and stress-response pathways, and its dysregulation contributes to disease processes related to proteotoxic and oxidative imbalance [131].

#### 7.6.9. NEK1

NEK1 (NIMA-related kinase 1) is a serine/threonine kinase, and LOF variants are among the more frequent genetic risk factors for ALS. It is located on chromosome 4q33 and expressed in multiple tissues, including motor neurons [132]. In healthy cells, *NEK1* contributes to multiple homeostatic processes, including primary cilia formation, DNA damage response, microtubule stability, and nucleocytoplasmic transport. ALS-associated *NEK1* variants impair ciliogenesis, as patient-derived fibroblasts carrying LOF or splice-site variants exhibit significantly reduced primary cilia frequency and length, with abnormal morphology. These variants also weaken the DNA damage response following genotoxic stress. *NEK1*-deficient cells fail to resolve γH2AX foci, show diminished Chk1 phosphorylation, and exhibit increased caspase-3 activation, indicating defective DNA repair and higher susceptibility to apoptosis [133]. Moreover, in motor neuron models, reduction or mutation of *NEK1* disturbs microtubule homeostasis and nuclear import: *NEK1* phosphorylates tubulin and importin-β1, and its deficiency causes instability of microtubules, mislocalization of nuclear transport factors, and impaired nucleocytoplasmic transport, all of which disrupt neuronal structural integrity and trafficking [132].

Clinically, *NEK1* LOF variants are estimated to account for approximately 2–3% of fALS and sALS cases, with patients often showing earlier onset and faster disease progression compared with non-carriers [134,135]. Population-based analyses have also revealed distinct ethnic variation in mutation frequency, with European cohorts showing higher rates of LOF variants than Asian populations [135].

These findings highlight NEK1 as a multifunctional kinase whose disruption compromises several homeostatic pathways—including ciliary signaling, DNA repair, and cytoskeletal integrity—ultimately predisposing neurons to degeneration. Its broad role across these networks makes NEK1 a promising target for future therapeutic interventions in ALS [133].

#### 7.6.10. ATXN2

The ATXN2 gene harbors a polyglutamine repeat region, and intermediate-length expansions of this region are among the strongest genetic risk factors for ALS [136]. In models combining patient-derived motor neurons with engineered mice harboring ATXN2 expansions (e.g., Q33), these expansions amplify TDP-43 toxicity and pathology, leading to defects in stress granule dynamics, neurite integrity, and neuronal electrophysiology. 

These pathogenic changes manifest alongside disruptions in metabolic and immune pathways. Transcriptome analyses reveal dysregulation of genes involved in oxidative phosphorylation, mitochondrial function, lipid metabolism, and inflammatory responses in both neurons and glial cells. Functionally, motor neurons with ATXN2 intermediate expansions show reduced basal and maximal mitochondrial respiration, indicating bioenergetic dysfunction [136].

In vivo, crossing ATXN2-expanded mice with TDP-43 transgenic models exacerbates motor deficits, neuromuscular junction alterations, and neurodegeneration. Together, these data show that ATXN2 expansions disrupt cellular homeostasis in ALS by perturbing mitochondrial energy metabolism, stress response systems (e.g., stress granules), and immunometabolic signaling, thereby pushing vulnerable motor neurons toward degeneration [136].

### 7.7. Gene Therapy for ALS

Gene therapy gained significant attention following the clinical approval of the first novel recombinant Ad-p53 gene therapy for head and neck squamous cell carcinoma in 2003 [137]. Since then, these therapies have represented a breakthrough across multiple fields, offering potential cures for debilitating conditions that remain untreatable with conventional methods. These include various cancers, muscular dystrophies, hemoglobinopathies, retinal dystrophies, and many others [138]. Up until 2024, the number of registered gene therapy clinical trials reached approximately 3900 globally [139].

The groundbreaking success of Zolgensma (onasemnogene abeparvovec), a gene therapy for pediatric SMA approved in 2019, has fueled intensive efforts to develop similar therapies for ALS [104,140]. These efforts culminated in the 2023 approval of QALSODY (tofersen), the first gene therapy indicated for patients with SOD1-mutant [141]. Despite this notable success, several major challenges remain before such therapies can be extended to the wider ALS population.

First, unlike the monogenic nature of SMA, which is primarily caused by homozygous loss or *SMN1* gene mutations, the genomic landscape of ALS is highly heterogeneous, involving multiple loci and pathogenic mechanisms, as previously described. This restricts the therapeutic indication of single-gene–targeted approaches. In the instance of Qalsody, approximately 98% of ALS patients are not eligible for the drug because SOD1 mutations account for only a small fraction of cases [142].

Second, most patients with ALS carry polygenic combinations of common and rare variants, or no identifiable pathogenic variants at all. This highlights the need for further genomic and mechanistic investigations to define disease etiology. To address this, several strategies have been employed to develop novel ALS therapies, including antisense oligonucleotides (ASOs), RNA interference (RNAi), or gene-editing tools like Clustered Regularly Interspaced Short Palindromic Repeats (CRISPR) systems [143].

### 7.8. Antisense Oligonucleotides (ASOs)

ASOs are a class of RNA-based therapeutics designed to modulate gene expression at the transcriptional level. These are short, synthetic strands of DNA or RNA, typically 8 to 50 nucleotides long, that bind specifically to target messenger RNAs (mRNAs) in cells [144]. Upon binding, they recruit the Ribonuclease H enzyme, which detects the RNA–RNA or RNA-DNA duplex formed by the ASO and its target mRNA, and then cleaves the mRNA. This targeted degradation reduces transcript stability and, in turn, decreases the production of the associated protein [145].

ASOs have shown significant therapeutic potential for treating genetic and acquired diseases, particularly cancer. Although they do not naturally cross the blood–brain barrier, intrathecal administration of ASOs allows for their widespread distribution throughout the CNS, making them especially valuable for treating neurological conditions. To date, ASO has been used in approved gene therapies for SMA and Duchenne muscular dystrophy, and more recently for ALS with Qalsody [146].

In ALS, Qalsody mediates the degradation of SOD1 mRNA, thereby significantly reducing neurofilament light chain levels, a key biomarker of neurodegeneration strongly associated with patient survival. It is administered intrathecally at three initial 100 mg doses at 14-day intervals, followed by a maintenance dose every 28 days [147]. Notably, the primary endpoint of the phase III clinical trial was not met. However, a meta-analysis including two randomized control trials, five cohort studies, and five case series and case reports demonstrated a significantly slower decline in ALS Functional Rating Scale-Revised scores in the intervention arm compared to placebo [148]. Finally, further trials are required to confirm long-term clinical benefits [147].

ASOs were tested on other targets, such as *C9orf72*, which showed no clinical benefit compared with placebo, leading to the abandonment of the clinical trial in 2024 [149]. Similarly, using ASO to target *ATXN2* demonstrated a promising 35-month survival benefit in a mouse model of TDP-43 proteinopathy [150], but no reduction in neurodegeneration biomarkers or change in clinical outcomes in humans (trial number NCT04494256) [151]. Further ongoing human trials utilizing ASO are elaborated in Table 3.

### 7.9. RNA Interference (RNAi)

Another gene therapy approach utilizes RNAi to silence the pathologic expression of disease-casing genes via the use of small RNA molecules, such as small interfering RNA (siRNA) or microRNA (miRNA), aiming to prevent protein translation. Viral vectors have been widely used in RNAi, particularly adeno-associated virus (AAVs) [152]. In ALS, this approach has recently been shown to increase the survival of TAR4/4 ALS mice by 50% by RNAi targeting *ATXN2* gene in AAV packaging [153]. This approach is an alternative to the disappointing clinical results of using ASO to target *ATXN2, as* mentioned above. One area to improve RNAi is allele-specific silencing, which may reduce mutant transcripts while sparing wild-type expression [154]. Currently, there are three ongoing clinical trials utilizing RNAi targeting SOD1 transcript, elaborated in Table 3.

### 7.10. Clustered Regularly Interspaced Short Palindromic Repeats (CRISPR)

Lastly, the CRISPR platform is used for in vivo DNA and mRNA repair, along with CRISPR-associated protein (Cas) complexes. This allows for precise genomic editing, replacement, or introduction of new gene fragments that can counteract the effects of the disease. One of the most common CRISPR systems is CRISPR-Cas9, in which a designed guide RNA binds to the desired region and guides the endonuclease Cas9 to create a double-stranded break, which is repaired by either non-homologous end joining or homology-directed repair, depending on whether a repair template has been introduced during repair [155,156]. Extensive preclinical work has investigated the efficiency of CRISPR-Cas systems in repairing mutations in ALS genes, including *C9orf72*, *TDP-43*, *SOD1*, *hSOD1*, and *MATR3*. So far, however, there are no CRISPR-based ALS clinical trials [157].

In ALS, preclinical studies have shown that excision of the C9orf72 hexanucleotide repeat expansion using CRISPR-Cas9 decreases RNA foci and dipeptide repeat accumulation in patient-derived pluripotent stem cells induced to become motor neurons [158]. Furthermore, the use of gene-silencing Cas13 and Cas7-11 enzymes prevented TDP-43 protein aggregation and increased the survival of TAR4/4 mice, a TDP-43 proteinopathy model [159].

Aside from clinical efficacy, several challenges arise in the clinical translation of gene therapy, including safety, ethical use, timing of intervention, accessibility, and high cost. Regarding safety concerns, administering high doses of AAV therapy can cause hepatotoxicity, as high viral load is required to achieve the desired transduction efficiency [160]. Moreover, off-target cuts by CRISPR systems can lead to unpredictable, deleterious genomic effects, such as oncogenesis [161]. This risk can be minimized through efficient guidance designs, genotoxicity evaluation in preclinical models, and long-term follow-up in clinical trials.

For the timing of treatment, it is vital to consider disease progression, as patients may be eligible to enroll in some gene therapy trials on a compassionate basis in late stages, which can represent a confounder if the treatment misses a potential early therapeutic window before progression [162].

Ethical considerations for gene therapies are unique. For instance, the irreversible nature of gene therapy interventions, as opposed to many conventional treatments, coupled with the unknown duration of benefit, affects the risk-benefit analysis. Additionally, the long-term follow-up for gene therapy trials might be affected by children’s refusal to consent once they reach adulthood [162].

Lastly, the linked issues of accessibility and cost constitute a major hurdle to the fair distribution of gene therapies. Qalsody, the approved gene therapy for ALS, is priced at approximately 310,000 USD for the first year, and 270,000 annually for subsequent years [163].

Pharmaceutical companies justify the high cost by the complex preparation and delivery systems, as well as the infrequent dosing for a small number of patients, compared with major chronic diseases that require continuous medication use for large numbers of patients. Not all healthcare systems reimburse such treatments above a ceiling to preserve funds for the broader population. As a result, this leaves a segment of patients with rare diseases ineligible for potentially life-saving gene therapies. This disparity in healthcare funding provision stems from the underlying ethical approach to fund allocation, whether utilitarian, maximizing benefits for the majority by sacrificing the needs of the minority, or non-utilitarian, ensuring no one is left behind at the expense of the majority [164].

To conclude, these advances in gene therapy approaches, such as ASOs, RNAi, and CRISPR-based editing, are forming a new paradigm in ALS treatment. Despite the ongoing challenges in drug delivery, long-term durability, and genomic heterogeneity of ALS, these approaches signify a shift from experimental trials toward personalized gene therapy capable of delaying onset, slowing progression, and potentially preventing ALS in genetically predisposed individuals.

## 8. Conclusions

In conclusion, this review demonstrates that ALS is a complex multisystem neurodegenerative disorder, arising from mitochondrial dysfunction, proteinopathies, altered redox status, glutamate-induced toxicity, failures in axonal transport, and dysfunction in immune cells. Clinically, this complexity manifests through various phenotypes and distinct disease pathways. Differences in disease across regions further suggest that ALS comprises overlapping biological subtypes rather than a single disease entity.

Recent advances in genomics have classified ALS into distinct molecular groups. Mutations in key genes such as *SOD1*, *C9orf72*, *TARDBP*, and *FUS* have enhanced our understanding of the disease and provided targets for novel treatments, including ASOs, RNAi, and CRISPR-based editing. However, transitioning from promising preclinical results to clinical use is challenging due to difficulties in target selection, treatment delivery, and determining the optimal timing and dose of therapies.

Recent research also suggests that the gut–brain axis may play a vital role in disease progression, but therapies targeting the microbiome are still in the early experimental stage. Ultimately, changing the natural course of ALS depends on a personalized multimodal approach. This includes early diagnosis using novel biomarkers, therapies targeting specific genotypes, systemic neuroprotective strategies, and gene-editing technologies. Coordinated global efforts that combine molecular classification, flexible clinical trials, and equitable access to genetic and treatment resources hold the most tremendous potential to transform ALS from a fatal condition into one that can be treated and possibly prevented in the near future.

## Figures and Tables

**Table 1:** Gold Coast vs. Revised El Escorial Criteria.

Feature	Gold Coast Criteria (2019) [39]	Revised El Escorial Criteria (1998, Revised 2000) [34]
Purpose	Simplified and inclusive criteria to enable earlier ALS diagnosis and cut down the diagnostic delay.	Designed for research trials. It ensures diagnostic certainty, but it is more restrictive.
Core Diagnostic Requirement	Progressive motor impairment with UMN and LMN dysfunction in at least one region, or lower motor neuron dysfunction in at least two regions. Exclusions have been ruled out.	UMN and LMN signs in various anatomical areas require progression and the exclusion of similar conditions.
Regions Required	Minimum: one region (UMN + LMN) or two regions (LMN only).	Multiple regions; stratified diagnostic certainty (Definite, Probable, Possible ALS).
Use of EMG	Supportive, but not required, if clinical findings are enough.	Strong focus on EMG to confirm LMN involvement if it is not clinically clear.
Certainty	ALS vs. Non-ALS	Multi-tier: Definite, Probable, Probable Laboratory supported, Possible.
Advantages	-Earlier diagnosis -Higher sensitivity -Simpler for clinical use	-High specificity, reliable for clinical trials -Well-established global standard
Disadvantages	-Slightly lower specificity—Less validated in long-term multi center trials	-Often delays diagnosis (10–16 months typical) -Confusing categories for patients/families

**Table 2:** Cytokine Dysregulation in ALS.

Cytokine	Type	Biological Role	ALS Relevance
IL-6	Pro-inflammatory	Activates astrocytes, promotes chronic inflammation	Elevated in serum, CSF, and spinal cord; correlates with progression [18]
IL-1β	Pro-inflammatory	Inflammasome signaling and excitotoxicity	Increased in ALS tissue; linked to motor neuron death [19]
TNF-α	Pro-inflammatory	Induces apoptosis and glial activation	Elevated in patient biofluids; worsens neurodegeneration [18,19]
IL-2	Immunoregulatory	Maintains Regulatory T Cell homeostasis	Elevated in serum; IL-2 therapy improves Regulatory T Cell function [19]
IL-10	Anti-inflammatory	Suppresses immune activation	Higher levels slow progression: overexpression prolongs survival in mouse models [20,21]
IFN-γ	Primarily pro-inflammatory; may exert protective effects in specific contexts (dose, timing dependent)	Modulates T cell and microglial responses; downstream of IL-18 singling	Elevated in CSF/serum; linked to faster progression but may be protective in some contexts [22,23]

**Table 3:** Summary of recent ALS clinical trials starting from the year 2020 that use both ASO and RNAi approaches. *ALS* amyotrophic lateral sclerosis, *FTD* frontotemporal dementia, *ASO* antisense oligonucleotide, *siRNA* small interfering RNA, *miRNA* microRNA.

Gene Therapy Approach	Target Gene	Drug Name	Trial Phase	Trial Design	Number of Participants	Trial Status	Estimated Completion Date	Trial Record
ASO	*FUS*	ION363 (Ulefnersen)	Phase 3	Randomized, double-blinded	89 (actual)	Active, not recruiting	March, 2028	NCT04768972
ASO	*ATXN2*	BIIB105	Phase 1/2	Randomized, triple-blinded	99 (actual)	Terminated	August, 2024	NCT04494256
ASO	*C9orf72*	WVE-004	Phase 1/2	Single-group, open-label	8 (actual)	Terminated	June, 2023	NCT05683860
RNAi (miRNA)	*SOD1*	AMT-162	Phase 1/2	non-randomized, open-label	20 (estimated)	Active, not recruiting	June, 2031	NCT06100276
RNAi (siRNA)	*SOD1*	RAG-17	Phase 1	Randomized, double-blinded	32 (estimated)	Recruiting	April, 2026	NCT06556394
RNAi (siRNA)	*SOD1*	ALN-SOD	Phase 1	Randomized, quadruple-blinded	42 (estimated)	Recruiting	April, 2029	NCT06351592

## Data Availability

Not applicable.
